# A flexible transoral swab sampling robot system with visual-tactile fusion approach

**DOI:** 10.3389/frobt.2025.1520374

**Published:** 2025-03-19

**Authors:** Jiaxiang Dong, Peng Li, Quanquan Liu, Qi Liu, Chunbao Wang, Xuezhi Zhao, Xiping Hu

**Affiliations:** ^1^ School of Mechanical and Automotive Engineering, South China University of Technology, Guangzhou, China; ^2^ School of Mechanical and Electrical Engineering and Automation, Harbin Institute of Technology (Shenzhen), Shenzhen, China; ^3^ Artificial Intelligence Research Institute & Guangdong-Hong Kong-Macao Joint Laboratory, Shenzhen MSU-BIT University, Shenzhen, China; ^4^ Department of Neurology, The First Affiliated Hospital of Shenzhen University, Shenzhen, China; ^5^ School of Mechanical and Electrical Engineering, Guangdong University of Science and Technology, Dongguan, China

**Keywords:** transoral swab manipulation, automatic sampling, flexible robot, visual-tactile fusion, deep learning

## Abstract

A significant number of individuals have been affected by pandemic diseases, such as COVID-19 and seasonal influenza. Nucleic acid testing is a common method for identifying infected patients. However, manual sampling methods require the involvement of numerous healthcare professionals. To address this challenge, we propose a novel transoral swab sampling robot designed to autonomously perform nucleic acid sampling using a visual-tactile fusion approach. The robot comprises a series-parallel hybrid flexible mechanism for precise distal posture adjustment and a visual-tactile perception module for navigation within the subject’s oral cavity. The series-parallel hybrid mechanism, driven by flexible shafts, enables omnidirectional bending through coordinated movement of the two segments of the bendable joint. The visual-tactile perception module incorporates a camera to capture oral images of the subject and recognize the nucleic acid sampling point using a deep learning method. Additionally, a force sensor positioned at the distal end of the robot provides feedback on contact force as the swab is inserted into the subject’s oral cavity. The sampling robot is capable of autonomously performing transoral swab sampling while navigating using the visual-tactile perception algorithm. Preliminary experimental trials indicate that the designed robot system is feasible, safe, and accurate for sample collection from subjects.

## 1 Introduction

Respiratory epidemic diseases, such as Coronavirus Disease 2019 (COVID-19) and seasonal influenza, pose significant threats to human health. Identifying infected individuals and isolating them from others is an effective strategy to mitigate the spread of these diseases. A commonly used diagnostic method is the nucleic acid test conducted through oropharyngeal (OP) swab sampling (Chen et al., 2020). Nucleic acid sampling continues to be a routine procedure at key locations, including customs, to prevent the transmission of respiratory diseases. Conventionally, sampling is manually performed by healthcare professionals (Kim et al., 2020). The quality of sampling, which is closely linked to the sampling position and sample volume, heavily depends on the operator’s skill, leading to inconsistencies and potential misdiagnoses (Li et al., 2020). Additionally, healthcare workers performing swab sampling are at risk of exposure, as close contact is a primary mode of virus transmission (Jin et al., 2020). On the other hand, in response to the insufficient nucleic acid testing capacity during the pandemic, self-sampling has emerged as a feasible option (Boum et al., 2021). Although this method helps reduce population movement and lowers the risk of virus transmission, it also presents some challenges, such as improper sampling techniques that may result in poor sample quality, difficulties in implementation for certain populations, and various factors that may influence the testing process (Lindner et al., 2021). Therefore, implementing non-contact sampling methods through teleoperation is an alternative strategy to minimize person-to-person contact, improve the consistency of sample quality, and protect operators by eliminating direct interaction with the testee (Feizi et al., 2021).

The typical teleoperated sampling method utilizes a master-slave configuration, where the operator maneuvers a master handle to remotely control a slave robot for sampling operations (Liu et al., 2022; Li et al., 2021; Hu et al., 2022). This approach helps to protect the operator from virus exposure resulting from person-to-person contact, however, it presents several challenges for precise samplings, such as eye-hand coordination issues, lack of immersion, and high workload (Zhong et al., 2020; Naceri et al., 2021). To address these limitations, recent efforts have focused on developing unmanned sampling robots that integrate visual and tactile feedback and robotic control. These systems aim to reduce dependency on human intervention while enhancing consistency in sampling quality (Huang et al., 2022; Sun et al., 2022). For example, image processing modules are commonly employed to guide sampling tasks, improving both accuracy and reliability. Furthermore, employing flexible and compact manipulators to facilitate transoral nucleic acid sampling is a promising solution to improve the automation of the sampling process and reduce the need for healthcare professional involvement. A low-cost, miniature robot with an active end-effector, a passive positioning arm, and a detachable swab gripper equipped with integrated force sensing capabilities has been introduced to assist with nasopharyngeal swab sampling (Wang et al., 2021). The system allows for large-range movement of the end effector through manual adjustment of the 6-DOF passive arm by medical personnel or the testee. Subsequently, the active end effector performs the swab collection task through translational and rotational movements. While the design offers advantages in cost and assembly simplicity, its 2-DOF end-effector severely limits mobility, particularly when handling nasal cavities of varying shapes and sizes. This constraint often leads to insufficient flexibility and positional inaccuracy during sampling. To overcome such challenges, researchers have explored continuum robots, such as concentric tube robots and tendon-driven systems, which demonstrate potential in delicate surgical tasks. For example, Webster’s research has revealed the potential of concentric tube robots in performing precise movements in constrained environments, such as during colorectal cancer resection surgeries (Dang et al., 2024). Morimoto, on the other hand, has assessed the feasibility of concentric tube robots for micro-laryngeal surgery, leveraging their small size and strong navigation ability in confined spaces (Lin et al., 2024). However, manufacturing challenges still exist for concentric tube robots, including the high cost of pre-bent tube fabrication and significant uncertainty in the manufacturing process, making its challenging in large-scale deployment in certain application, such as transoral swab sampling. While tendon-driven robots offer high flexibility and adaptability (Dupont et al., 2022; Della Santina et al., 2023), the durability issues related to long-term operation of the tendons affect the robots’ stability and performance. Additionally, the complexity of real-time deformation computation and control of flexible actuators increases the time cost of sampling procedures. It is important to note that both types of robots are still in the research stage and have not yet matured.

Compared to existing methods, this paper proposes a flexible robotic system for automatic swab sampling, which employs a 4-DOF hybrid series-parallel mechanism and a 1-DOF linear motion module to achieve precise and flexible operation of the active end effector. The rigid hybrid mechanism is driven by double-screws, avoiding the nonlinear modeling errors caused by the use of flexible components, thereby significantly improving the system’s accuracy and responsiveness. Additionally, the system integrates visual and tactile feedback modules, enhancing the accuracy and ease of operation in the automated sampling process, thus alleviating the strain on medical resources. The main contributions of this work are as follows:

• A robotic system featuring a flexible mechanism for transoral swab sampling is introduced. Through a 4-DOF hybrid series-parallel mechanism with a dual-segment orthogonal planar bending configuration and a 1-DOF linear motion module, the system is capable of performing precise and flexible sampling operations within a confined space.
• An automatic identification algorithm for the posterior OP wall is developed, enabling precise navigation of the sampling position using a deep learning method.
• A visual-tactile fusion method for autonomous sampling is introduced, integrating visual image recognition and contact force feedback for enhanced operational accuracy.

The remainder of this paper is structured as follows: Section 2 describes the mechanical components of the robotic system and the visual-tactile fusion algorithm for position identification in autonomous sampling. Section 3 details the experimental results and discussion. Finally, Section 4 presents the conclusions.

## 2 Materials and methods

### 2.1 Mechanical components of the robotic system

**FIGURE 1:**
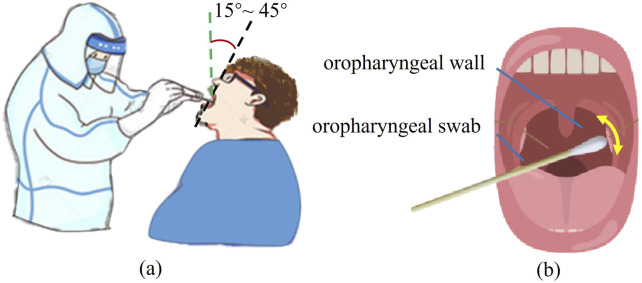
Transoral OP swab sampling. **(A)** Manual sampling scene. **(B)** Sampling practice with the OP swab.

The manual method for transoral OP swab sampling is depicted in Figure 1. In this procedure, the testee opens their mouth to expose the OP wall while tilting their head back at an angle of approximately 15°–45°. Simultaneously, the healthcare professional inserts an OP swab into the testee’s mouth and collects a nucleic acid specimen from the OP wall using a pendulum-like movement, guided by visual feedback and force perception through their eyes and hands, respectively.

**FIGURE 2:**
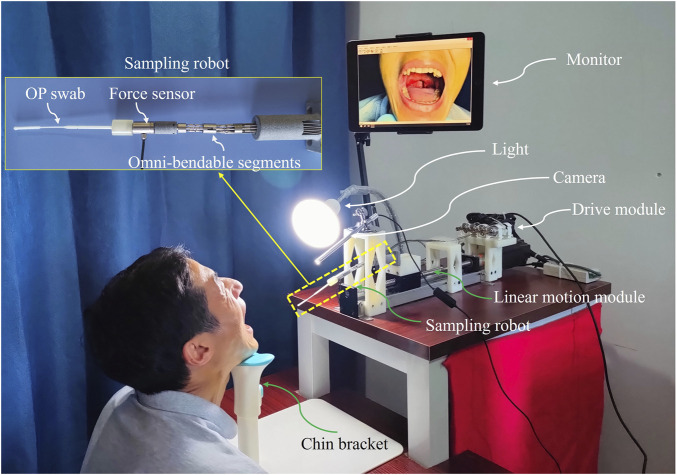
System overview of the sampling robotic system.

To replicate the nucleic acid sampling practices of medical workers, the proposed robotic system must provide dexterous manipulation along with visual and tactile force feedback. Based on these requirements, the proposed sampling robot comprises a linear motion module, a flexible series-parallel hybrid mechanism, and a visual-tactile fusion module. The flexible sampling mechanism, mounted on the movable platform of the linear motion module, enables straight reciprocating motion. A series-parallel hybrid mechanism is designed to achieve omnidirectional bending for the pose adjustment of the swab while providing tactile feedback through a force sensor mounted at the distal end. A camera captures an image of the testee’s OP with light assistance while the testee maintains an open-mouth position. A monitor displaying the camera’s live feed is placed in front of the testee to prompt them to expose their posterior pharyngeal wall as fully as possible. Flexible shafts are employed to connect the sampling mechanism to the drive module, serving as a power transmission medium that enables a compact and dexterous spatial layout (Liu et al., 2017). The overview of the OP swab sampling robotic system is presented in Figure 2.

### 2.2 Transoral sampling workspace analysis based on flexible mechanism

**FIGURE 3:**
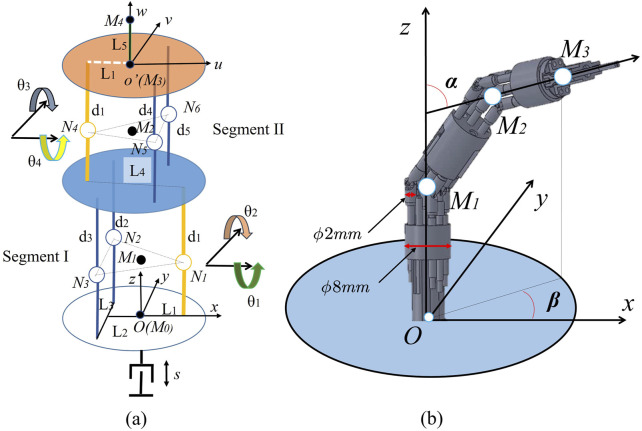
Nomenclature of the kinematics description for the sampling robot. **(A)** Kinematic skeleton frame of the robot. **(B)** Pose state in a polar coordinate system.

Similar to manual sampling manipulation, the sampling robot utilizes a linear motion module for large-scale movement and employs a flexible series-parallel hybrid mechanism for precise pose regulation. In this study, the series-parallel hybrid mechanism has a diameter of 8 mm and is composed of two bendable segments, each able to achieve a maximum omnidirectional bending angle of 45°. The nomenclature of the robot is depicted in Figure 3, and the kinematics parameters are detailed in Table 1.

**TABLE 1:** The kinematics parameter of the sampling robot.

i	$\theta_{x}$ (degree)	$\theta_{y}$ (degree)	$\theta_{z}$ (degree)	Δx (mm)	Δy (mm)	Δz (mm)	Range
1	0	0	0	L_1_ (2.4)	0	s + d_1_ (15)	0–100 mm
2	$\theta_{1}$	0	0	0	0	0	−45°–45°
3	0	$\theta_{2}$	0	0	0	0	−45°–45°
4	0	0	0	-L_4_ (4.8)	0	2d_1_ (30)	
5	0	$\theta_{3}$	0	0	0	0	−45°–45°
6	$\theta_{4}$	0	0	0	0	0	−45°–45°
7	0	0	0	L_1_ (2.4)	0	d_1_	
8	0	0	0	0	0	L_5_ (168)	

Where the kinematics parameters are noted as follows: M_i_ (i = 0, … ,4): feature point of the origin, the pivot point of the bent joint, and both ends of the distal part. N_i_ (i = 1, … ,5): pivot point of the universal joint. s: translational journey along axis z . α : polar angle of the distal of the robot under a spherical coordinate system. β : azimuthal angle of the distal of the robot under a spherical coordinate system. $\theta_{i}$ (i = 1, … ,4): the bending angle of the bendable segments along axis x or y . L_i_ (i = 1, … 5): L_1_ represents the deviation between the support rod and the origin of the coordinate system along axis x . L_2_ represents the deviation between the active rod and the origin of the coordinate system along axis x . L_3_ represents the distance between two active rods. L_4_ represents the distance between two support rods. L_5_ represents the length of the distal part. d_i_ (i = 1, … 4): d_1_ represents the length of the support rod. d_2_ and d_3_ represent the length of the active rods in segment I, respectively. d_4_ and d_5_ represent the length of the active rods in segment II, respectively. Each of these rods is designed with a uniform diameter of 2 mm.

**FIGURE 4:**
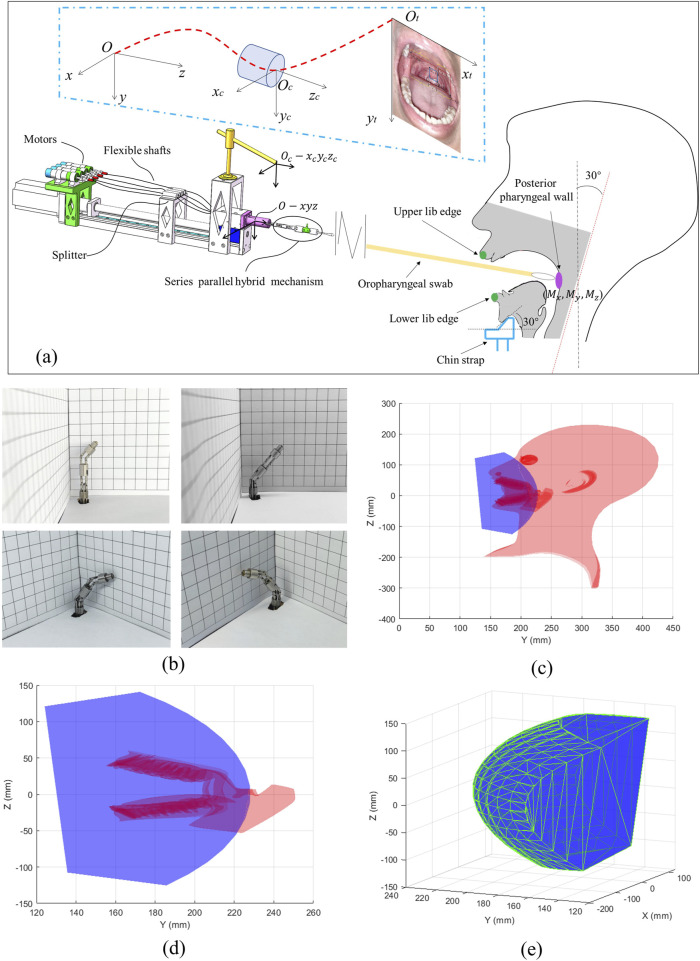
Workspace of the flexible sampling robot. **(A)** Spatial configuration between the sampling robot and the testee’s oral cavity. **(B)** Illustration of the bending movement facilitated by the series-parallel hybrid mechanism. **(C)** Positioning of the workspace of the distal end of the OP swab relative to the testee’s oral cavity. **(D)** Enlarged partial view of **(C)**. **(E)** Workspace of the distal end of the OP swab.

During the sampling process, each testee positions their chin on a fixed chin strap, pressing it firmly against an inclined plane tilted at a 30° angle to ensure their head is raised with a backward angle of 30°. The testee then opens their mouth to expose the posterior pharyngeal wall as much as possible, preparing for automatic sampling with robotic assistance. To reduce the risk of cross-contamination, the testee will be provided with a sterile paper, shaped to fit the chin strap, which they will place before the sampling procedure. Additionally, healthcare staff will regularly clean and disinfect the chin strap to ensure hygiene and minimize potential transmission risks. Typically, the mouth opening of an adult measures approximately 50 mm in height. The OP swab head is inserted into the testee’s oral cavity and directed toward the oropharyngeal wall through joint motion facilitated by the linear motion module and the series-parallel hybrid mechanism. In this study’s sampling robot platform, the camera captures a two-dimensional (2D) image without depth information. Therefore, the position of the sampling point in $o_{t}$ - $x_{t}y_{t}$ is identified using visual feature recognition, while the position along the z-axis is determined through tactile force sensing feedback. The details of the position recognition process using the visual-tactile fusion algorithm are discussed in Section 2.3. The relationship among the world coordination system o - xyz , the camera coordination system $o_{c}$ - $x_{c}y_{c}z_{c}$ , and the sampling point coordination system $o_{t}$ - $x_{t}y_{t}$ is shown in Figure 4A.

In order to calculate the workspace of the sampling robot, we define a homogenous transformation matrix as

Ti−1 i=Rx|y|zT01 (1)

where

$$\begin{array}{c} R_{x}=\left[\begin{array}{ccc} 1 & 0 & 0\\ 0 & \cos\theta_{x} & -\sin\theta_{x}\\ 0 & \sin\theta_{x} & \cos\theta_{x} \end{array}\right],R_{y}=\left[\begin{array}{ccc} \cos\theta_{y} & 0 & \sin\theta_{y}\\ 0 & 1 & 0\\ -\sin\theta_{y} & 0 & \cos\theta_{y} \end{array}\right],\\ R_{z}=\left[\begin{array}{ccc} \cos\theta_{z} & -\sin\theta_{z} & 0\\ \sin\theta_{z} & \cos\theta_{z} & 0\\ 0 & 0 & 1 \end{array}\right],T=\left[\begin{array}{c} P_{x}\\ P_{y}\\ P_{z} \end{array}\right] \end{array}$$

and $\theta_{x}$ , $\theta_{y}$ , $\theta_{z}$ represent rotating angles along axes x , y , z , respectively. $P_{x}$ , $P_{y}$ , $P_{z}$ represent coordinates along axes x , y , z , respectively.

By substituting the parameters from Table 1 into Equation 1, the forward kinematic chain can be expressed as

T08=∏i=18Ti−1 i (2)

Based on Equation 2 describing the kinematic transformation, the coordinate of the distal of the OP swab is given by

PM4=T1,408T2,408T3,408T (3)

Therefore, the workspace of the sampling robot can be determined by substituting the kinematic parameters from Table 1 into Equation 3. The calculated workspace is illustrated in Figure 4.

Suppose the coordinate and orientation of sampling point M as

PM=MxMyMz,R oM=uxvxwxuyvywyuzvzwz (4)

where $P_{M}$ represents the coordinate of OP swab tip, and RoM represents the orientation matrix of OP swab in o - xyz coordination system; they can be obtained via Bryan angle transformation. As shown in Equation 5, the coordinate of point $o^{\prime }$ can be calculated as

Po′=PM+RoM•PΔ (5)

where $P_{o^{\prime }}$ represents the coordinate of point $o^{\prime }$ in the o - xyz coordination, and $P_{\Delta }={\left[\begin{array}{ccc} 0 & 0 & -L_{5} \end{array}\right]}^{T}$ .

Combining Table 1 and (Equation 1), vector $\overset{\to }{M_{1}M_{2}}$ and $\overset{\to }{M_{2}M_{3}}$ can be computed as

$$\begin{array}{cc} \overset{\to }{M_{1}M_{2}} & =\left[\begin{array}{c} 2d_{1}sin\theta_{2}\\ -2d_{1}cos\theta_{2}sin\theta_{1}\\ 2d_{1}cos\theta_{1}cos\theta_{2} \end{array}\right],\\ \overset{\to }{M_{2}M_{3}} & =\left[\begin{array}{c} d_{1}cos\theta_{4}\sin\left(\theta_{2}+\theta_{3}\right)\\ d_{1}\left(-cos\theta_{1}sin\theta_{4}-sin\theta_{1}cos\theta_{4}\cos\left(\theta_{2}+\theta_{3}\right)\right.\\ d_{1}\left(-sin\theta_{1}sin\theta_{4}+cos\theta_{1}cos\theta_{4}\cos\left(\theta_{2}+\theta_{3}\right)\right. \end{array}\right] \end{array}$$ (6)

In this study, the series-parallel structure is configured to conform to an arc shape. Therefore, the polar angle of the vector $\overset{\to }{M_{1}M_{2}}$ , $\overset{\to }{M_{2}M_{3}}$ is α /2 and α respectively. Based on Equation 6, the relation between $\theta_{i}$ (i = 1, … ,4) and α , β can be expressed by

$$cos\frac{\alpha }{2}=2cos\theta_{1}cos\theta_{2}$$ (7)

$$tg\beta =-sin\theta_{1}\frac{cos\theta_{2}}{sin\theta_{2}}$$ (8)

$$cos\alpha =-sin\theta_{1}sin\theta_{4}+cos\theta_{1}cos\theta_{4}cos\left(\theta_{2}+\theta_{3}\right)$$ (9)

$$tg\beta =\frac{-cos\theta_{1}sin\theta_{4}-sin\theta_{1}cos\theta_{4}cos\left(\theta_{2}+\theta_{3}\right)}{cos\theta_{4}sin\left(\theta_{2}+\theta_{3}\right)}$$ (10)

Based on the combined use of (Equations 7, 8), $\theta_{i}$ (i = 1,2) can be calculated as

$$\theta_{2}=arcsin\frac{sin\left(\alpha /2\right)}{\sqrt{1+tg^{2}\beta }},\theta_{1}=arcsin\left(-tg\beta \cdot tg\theta_{2}\right)$$ (11)

Due to $\theta_{3}$ and $\theta_{4}$ coupling with each other, an iterative algorithm is established to calculate $\theta_{3}$ and $\theta_{4}$ to reduce the difficulty of the solving process. Combining Equations 9-11, the Pseudo code is presented in Table 2.

**TABLE 2:** Pseudo code for angle calculation of $\theta_{3}$ and $\theta_{4}$

Calculate angle $\theta_{3}$ and $\theta_{4}$ from ( α , β , $\theta_{1}$ , $\theta_{2}$ )
1. Input: ( α , β , $\theta_{1}$ , $\theta_{2}$ )
2. for $\theta_{3}$ = −45°,…, 45° do
3. $\theta_{4\text{\_}1}=arctan\frac{-sin\theta_{1}cos\left(\theta_{2}+\theta_{3}\right)-tg\beta sin\left(\theta_{2}+\theta_{3}\right)}{cos\theta_{1}}\leftarrow$ (Equation 10)
$\theta_{4\text{\_}2}=arccos\frac{cos\alpha cos\theta_{1}}{cos\left(\theta_{2}+\theta_{3}\right)+tg\beta sin\theta_{1}sin\left(\theta_{2}+\theta_{3}\right)}\leftarrow$ (Equations 9, 10)
4. $\alpha^{\prime }=arc\cos\left(-sin\theta_{1}sin\theta_{4\text{\_}1}+cos\theta_{1}cos\theta_{4\text{\_}1}cos\left(\theta_{2}+\theta_{3}\right)\right)\leftarrow \left(\theta_{1},\theta_{2},\theta_{3},\theta_{4\text{\_}1}\right)$
$\beta^{\prime }=arctan\left(\frac{-cos\theta_{1}sin\theta_{4\text{\_}1}-sin\theta_{1}cos\theta_{4\text{\_}1}cos\left(\theta_{2}+\theta_{3}\right)}{cos\theta_{4\text{\_}1}sin\left(\theta_{2}+\theta_{3}\right)}\right)\leftarrow \left(\theta_{1},\theta_{2},\theta_{3},\theta_{4\text{\_}1}\right)$
$\alpha^{\prime \prime }=arccos\left(-sin\theta_{1}sin\theta_{4\text{\_}2}+cos\theta_{1}cos\theta_{4\text{\_}2}cos\left(\theta_{2}+\theta_{3}\right)\right)\leftarrow \left(\theta_{1},\theta_{2},\theta_{3},\theta_{4\text{\_}2}\right)$
$\beta^{\prime \prime }=arc\tan\left(\frac{-cos\theta_{1}sin\theta_{4\text{\_}2}-sin\theta_{1}cos\theta_{4\text{\_}2}cos\left(\theta_{2}+\theta_{3}\right)}{cos\theta_{4\text{\_}2}sin\left(\theta_{2}+\theta_{3}\right)}\right)\leftarrow \left(\theta_{1},\theta_{2},\theta_{3},\theta_{4\text{\_}2}\right)$
5. $f_{1\left(\theta_{1},\theta_{2},\theta_{3},\theta_{4\text{\_}1}\right)}=\left|\alpha -\alpha^{\prime }\right|+\left|\beta -\beta^{\prime }\right|,f_{2\left(\theta_{1},\theta_{2},\theta_{3},\theta_{4\text{\_}2}\right)}=\left|\alpha -\alpha^{\prime \prime }\right|+\left|\beta -\beta^{\prime \prime }\right|$
6. $\left(\theta_{1},\theta_{2},\theta_{3},\theta_{4}\right)\leftarrow min\left(f_{1\left(\theta_{1},\theta_{2},,\theta_{3},\theta_{4\text{\_}1}\right)},f_{2\left(\theta_{1},\theta_{2},,\theta_{3},\theta_{4\text{\_}2}\right)}\right)$
7. Output: optimal solution $\left(\theta_{1},\theta_{2},\theta_{3},\theta_{4}\right)$

By substituting the optimal solution into Equation 3, the translational journey s can then be calculated using the combined application of Equations 3, 4.

### 2.3 Visual-tactile fusion algorithm of position identification for autonomous sampling

**FIGURE 5:**
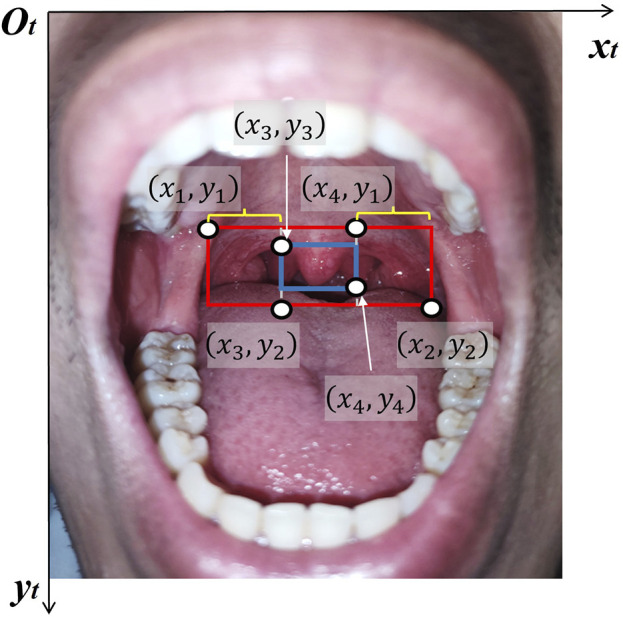
ROI coordinates calculation based on uvula and posterior pharyngeal wall.

The posterior pharyngeal wall is the recommended site for oropharyngeal swab sampling. As illustrated in Figure 1, the camera captures an image of the testee’s oral cavity after they open their mouth to expose the posterior pharyngeal wall. Since the image input into the computer is of a fixed size, the top left corner of the image is defined as the origin. The coordinates of the posterior pharyngeal wall relative to this origin are then calculated in the $o_{t}$ - $x_{t}y_{t}$ frame (Figure 5). To effectively identify the boundaries of the posterior pharyngeal wall, the features of the tonsil glands, posterior pharyngeal wall, and uvula are initially labeled. Subsequently, a recognition model for the posterior pharyngeal wall is developed using a deep learning algorithm. The algorithm steps are as follows.

#### 2.3.1 Dataset construction

**FIGURE 6:**
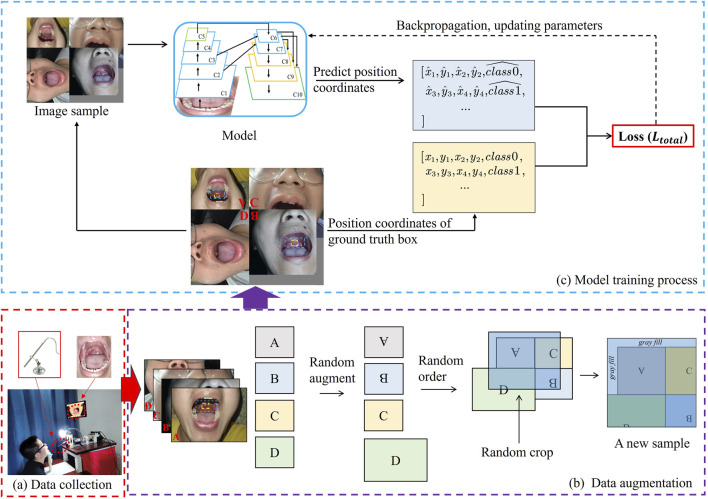
Procedures of training model. **(A)** Image data collection of the oral cavity. **(B)** Data augmentation using the input image. **(C)** Transfer learning algorithm for training model.

To address the challenge of boundary recognition, this study creates a dataset by collecting oral cavity images and manually annotating the boundary box labels for the 42 images. Two regions are identified: the blue box (uvula) and the red box (posterior pharyngeal wall), as shown in Figure 5. The required regional coordinates are determined based on the positions of these two regions. The coordinates obtained from the object detection model for the two target regions are represented by the blue box $\left(\left(x_{3},y_{3}\right),\left(x_{4},y_{4}\right)\right)$ and the red box $\left(\left(x_{1},y_{1}\right),\left(x_{2},y_{2}\right)\right)$ . Meanwhile, the coordinates of the Region of Interest (ROI), enclosed by braces, are calculated as $\left(\left(x_{1},y_{1}\right),\left(x_{3},y_{2}\right)\right)$ and $\left(\left(x_{4},y_{1}\right),\left(x_{2},y_{2}\right)\right)$ . The obtained oral cavity images are divided into two sets: the positive sample set and the negative sample set. In the positive sample set, the features of the tonsil glands, posterior pharyngeal wall, and uvula are labeled using a bounding box frame (Figure 5). Conversely, no labels are marked in the images of the negative sample set due to the absence of identifiable features. To enhance the adaptability of the recognition model to input images of the oral cavity in varying poses, Mosaic data augmentation techniques are applied to the input images prior to model training, including rotation, flipping, or randomly cropping, as shown in the Figure 6B. Then, multi-scale features of the posterior pharyngeal wall are used for training, and the model is optimized with adaptive anchor strategies to ensure dynamic recognition of feature zones of varying sizes.

#### 2.3.2 Parameterization and modeling

Yolov5 model is used for feature detection in this study, the loss function (Zheng et al., 2020; Jocher et al., 2021) is expressed as

$$L_{\mathrm{total}}=0.05L_{\mathrm{box}}+0.5L_{\mathrm{class}}+L_{\mathrm{confidence}}$$ (12)

where $L_{\mathrm{total}}$ means the total loss, $L_{\mathrm{box}}$ represents the loss between the prediction box and the ground truth box, $L_{\mathrm{class}}$ represents the class loss of the prediction box, $L_{\mathrm{confidence}}$ represents the confidence loss of the prediction box. They can be further expressed as Equation 13.

$$L_{\mathrm{box}}=1-IoU+\frac{\rho^{2}\left(b,b^{gt}\right)}{c^{2}}+\alpha v$$ (13)

where IoU means the Intersection of Union, the parameter v measures the consistency of aspect ratio, and parameter α is a positive trade-off parameter,

$$\begin{array}{cc} & v=\frac{4}{\pi^{2}}{\left(arctan\frac{w^{gt}}{h^{gt}}-arctan\frac{w}{h}\right)}^{2},\\ & \alpha =\frac{v}{\left(1-IoU\right)+v} \end{array}$$

The b and $b^{gt}$ denote the central points of the prediction box and ground truth box, ρ(⋅) is the Euclidean distance, and c is the diagonal length of the smallest enclosing box covering these two boxes.

Further, $L_{\mathrm{class}}$ , $L_{\mathrm{confidence}}$ can be calculated using Equation 14.

$$L_{\mathrm{class}},L_{\mathrm{confidence}}=\frac{1}{N}\overset{N}{\underset{i=1}{\sum }}\left(y_{i}•\log\left(\sigma \left(p_{i}\right)\right)+\left(1-y_{i}\right)•\log\left(1-\sigma \left(p_{i}\right)\right)\right)$$ (14)

The parameter $y_{i}$ is a binary label 0 or 1 of sample $x_{i}$ , and $p_{i}$ represents the probability of sample $x_{i}$ being predicted as a positive class. σ refers to the sigmoid function. The model training process is shown in Figure 6C.

The dataset of oral cavity images is divided into a training set (80%) and a test set (20%). All images are standardized to a size of 640* 640 pixels and then input into the Yolov5 model within the PyTorch environment. Stochastic Gradient Descent (SGD) and Automatic Mixed Precision (AMP) methods are employed to accelerate computation speed and reduce memory usage, enabling a significantly larger batch size. A learning rate schedule is implemented to dynamically adjust the learning rate parameter. The experimental results for the test set after running the training model for 300 epochs are presented in Table 3.

**TABLE 3:** The Effect of target detection model on detecting uvula and posterior oropharynx.

Class	Precision	Recall	mAP50	mAP
Uvula	0.891	0.824	0.857	0.522
Posterior oropharynx	0.99	0.842	0.979	0.626

As shown in Table 3, based on the training model, the average precision (mAP50) for uvula detection is 85.7%. Meanwhile, the mAP50 for posterior pharyngeal wall detection is 97.9%. The inference speed is 15.0 ms per image at a resolution of (640, 640), making it suitable for sampling navigation.

**FIGURE 7:**
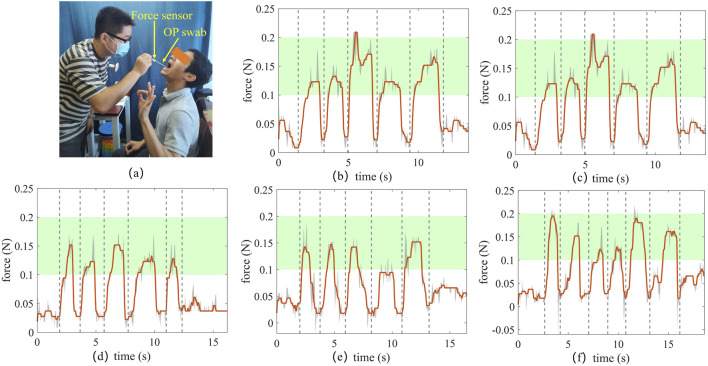
Force estimate experiment for OP swab sampling. **(A)** Experimental setup **(B–F)** Force measurement from five subjects with self-feeling the contact is not too hard to cause discomfort.

In this study, the sampling positions along the x and y-axes can be calculated using the aforedescribed visual processing algorithm. The disposable swab comprises a handle, a slender connecting rod, and a sampling head. The slender connecting rod has sufficient flexibility under lateral force, allowing a force-sensing capability along the axial direction of the connecting rod to be implemented to ensure sampling safety. A force sensor mounted at the distal end of the sampling robot is used to determine whether the head of the OP swab has reached the testee’s oropharyngeal wall. Since the goal is to obtain the axial contact force threshold along the Z-axis and to subjectively perceive the contact force threshold as a rough validation for the final contact force threshold, the contact force threshold assessment in this study was conducted through manual sampling. The experimental setup is shown in the Figure 7A. An OP swab and a rod are mounted on both sides of the tension/compression force sensor. a tester holds the rod and manually inserts the OP swab into a testee’s mouth to measure the contact force during swab sampling. Five subjects were invited to participate in this test. The testee raises their hand with an OK gesture when they feel that the contact force is appropriate after the swab contacts their oropharyngeal wall. The contact forces collected from OP swab sampling are shown in Figures 7B–F. The peaks in the contact force curve indicate that the tester has observed the testee’s OK gesture and ceased advancing the OP swab. As shown in Figure 7, all contact force peaks fall within the range of 0.1 N–0.2 N, with most centered around 0.15 N. At the same time, compared to previous studies (Sun et al., 2022) which reported a required sampling force threshold of at least 0.1 N for effective epithelial cell collection, the experimental results (0.1–0.2 N) fully meet this criterion while avoiding excessive forces that may cause discomfort. Therefore, a contact force threshold of 0.15 N is set to assess whether the OP swab advances appropriately during the sampling procedure.

The position of the sampling point in the xoy plane can be calculated using the visual processing algorithm, while the translational movement along the z-axis is determined through contact force feedback.

The application of the visual-tactile fusion method ensures that the OP swab accurately collects nucleic acid specimens while preventing excessive contact that could cause discomfort.

## 3 Experiments and discussion

We conducted experimental assessments to evaluate the effectiveness of the sampling robotic system in comparison to manual sampling procedures. A visual-tactile fusion module was developed to replicate the eye-hand coordination of testers during OP swab sampling.

### 3.1 Autonomous OP swab sampling

**FIGURE 8:**
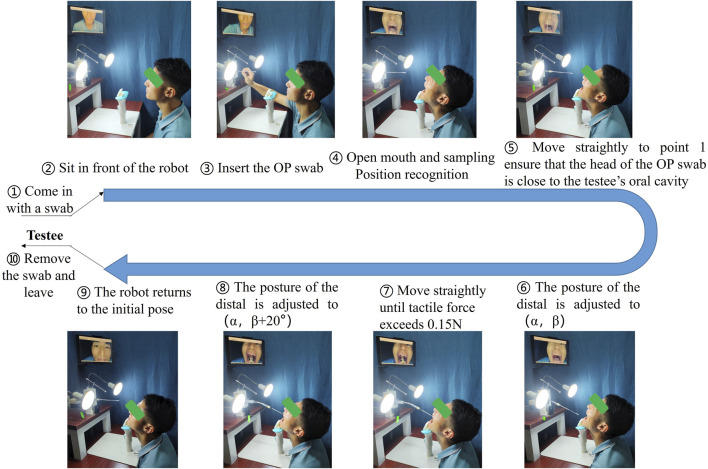
Experimental procedures.

Figure 8 illustrates the operation of the sampling robot during OP swab sampling. The detailed procedures are as follows: ① the sampling robot moves to its initial straight position (s = 0, $\theta_{i}$ = 0, i = 1, … ,4). ② A testee takes a disposable OP swab and sits in front of the sampling robot. ③ the testee inserts the OP swab into the distal holder of the sampling robot. ④ the testee leans their chin against the tilted chin strap and opens their mouth as wide as possible to expose the oropharyngeal wall while self-checking the monitor image suspended in front. The coordinates of the sampling point are calculated using the method described in Section 2.3. The polar angle α and azimuthal angle β of the flexible mechanism are subsequently computed based on the coordinates of the sampling point. ⑤ the translational joint advances forward and stops when the base of the sampling robot reaches fixed point 1, positioning the head of the OP swab near the testee’s oral cavity. ⑥ the distal of the sampling robot is adjusted to posture with parameters ( α , β ). ⑦ the translational joint moves forward until the head of the OP swab has contacted the oropharyngeal wall, then stops moving forward when the contact force exceeds 0.15 N. ➇ the distal of the sampling robot will perform an arcing trail to pose ( α , β +20°), which allows the head of the OP swab to collect enough sampling specimens. ➈ the sampling robot returns to the initial position. ➉ the testee removes the swab and leaves.

**FIGURE 9:**
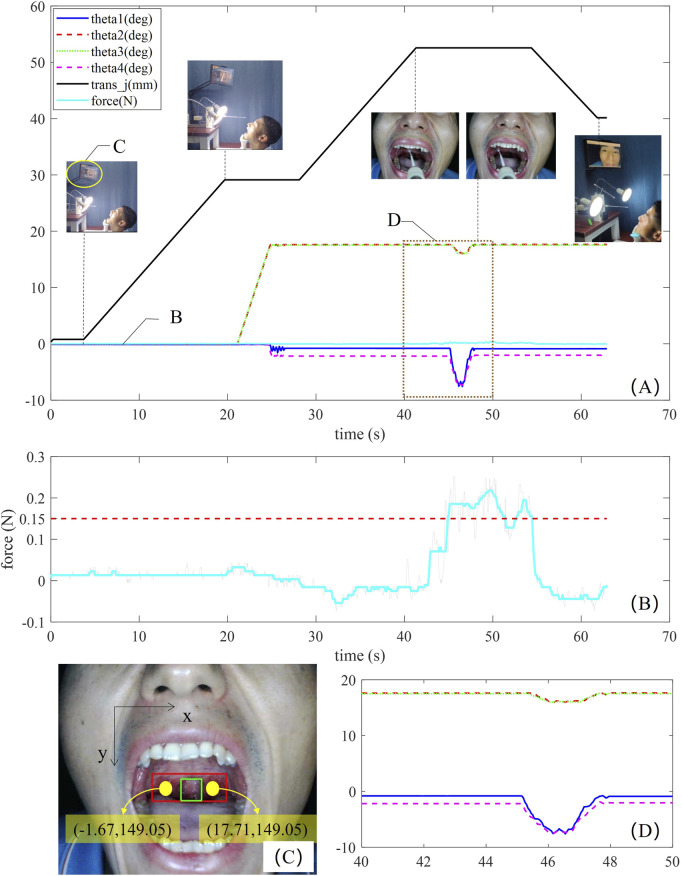
Experimental results. **(A)** Variation in the trace of the robot parameters during sampling manipulation. **(B)** Enlarged view of the tactile force curve. **(C)** Position recognition. **(D)** Angular variation in the trace of the robot joint as the OP swab performs the wiping movement on the posterior pharyngeal wall.

A volunteer was invited to participate in this experiment. The volunteer opens his mouth and self-checks the mouth pose on the monitor located at the front. Then, the zone of the posterior pharyngeal wall was identified, and the coordinates of the corner points were calculated by visual processing with a deep learning algorithm. The experimental results during sampling work are shown in Figure 9. The variation of a trace of the robot parameters during sampling operation is presented in Figure 9A, the meanings of labels from ④ to ➈ are the same as that of labels in Figure 8. In this sampling trail, the coordinates of the sampling point in the oral cavity were (−1.67, 149.05) mm and (17.71, 149.05) mm computed by the visual processing method (Figure 9C), then, the polar angle and the azimuthal angle of the sampling robot were calculated as (34.18°, 0.64°) at the left sampling point, which could be further used to resolve the bending angles of the series-parallel mechanism. The OP swab was inserted into the testee’s oral cavity after the distal posture had been adjusted to the angle of (34.18° and 0.64°), and stopped when the tactile force detection had exceeded 0.15 N. Then, the posture of the OP swab was driven to (34.18°, 20.64°) to collect more specimens, mimicking the sampling skill of the medical professional. The tactile force of the sampling robot was shown in Figure 9B, and the value was around 0.15 N during the whole sampling practice.

### 3.2 Discussion

Nucleic acid testing is an effective method for identifying individuals infected with COVID-19 or seasonal influenza. OP swab sampling is commonly used to collect specimens from the testee’s oral cavity. Despite stringent protective measures, medical staff remain at risk of infection due to the close contact nature of the procedure. In this study, we proposed an OP swab sampling robotic system comprising a series-parallel hybrid structure manipulator and a visual-tactile fusion module for autonomous sampling navigation. The system aims to minimize interaction between medical personnel and the testee, addressing safety concerns and improving sample quality consistency. A monitor positioned in front of the testee replicates the image of the oral cavity captured by the camera, allowing the testee to self-check their oral posture. A deep learning algorithm was developed to identify the sampling position in the posterior oropharynx. The preliminary trial demonstrated the feasibility of the robotic system for OP swab sampling, as shown in Figure 9. However, further research is needed to improve the system’s precision and adaptability in different clinical environments.

We designed a serial-parallel hybrid structure manipulator to perform posture adjustments for OP swab sampling. Compared to existing autonomous sampling systems that rely on limited DOF manipulators [such as the 2-DOF end effector in (Wang et al., 2021) and the 3-DOF end effector in (Hu et al., 2022)], our 4-DOF serial-parallel hybrid mechanism demonstrates superior adaptability to complex oral anatomies, achieving full workspace coverage in simulated oral cavities (Figures 4B–E). Additionally, the successful implementation of the automatic oropharyngeal swab experiment further validates the flexibility in posture adjustment provided by this hybrid mechanism. It can be configured into an arc shape by minimizing the deviation angle between the two bendable segments, and it can also be adjusted into a z shape by driving the two segments to bend in opposite directions. This enables the robotic system to adapt to a variety of oral configurations, reducing the potential for inaccurate sampling caused by rigid manipulators. At the same time, compared to serial-jointed end effectors (Hu et al., 2022; Wang et al., 2021; Li et al., 2021), the serial-parallel hybrid manipulator offers significant potential for OP swab sampling, even in scenarios involving obstacles such as an arched tongue or tooth occlusion. Although promising results were achieved, several challenges remain. For example, in the series-parallel hybrid mechanism, driven by flexible shafts, the rotational drive amount from the motor output is not perfectly transmitted to the distal end. Due to the elasticity and friction of the flexible shafts, one full rotation of the motor does not equivalently drive the distal segment of the flexible shafts to reach the expected drive amount, which makes the intermediate transmission model require more precise construction to ensure accurate system control. Additionally, the complexity of the system increases the time required for each test. While the duration is competitive with manual procedures, it may still vary depending on the testee’s cooperation and the complexity of the oral anatomy. Furthermore, to further optimize the system’s design and functionality, future research will also focus on user experience evaluations. This will include collecting feedback from medical personnel and testees to assess the system’s usability, ease of operation, and comfort during the testing process.

Traditional methods for detecting the posterior pharyngeal wall typically involve first detecting the face and oral cavity, followed by identifying the posterior pharyngeal region within the oral cavity (Sun et al., 2022). In this study, the developed recognition algorithm directly trains on multi-scale features of the posterior pharyngeal wall and is optimized using adaptive anchor strategies, ensuring adaptive recognition of feature regions of varying sizes. Mosaic data augmentation was applied to 42 original images to expand and construct the dataset, resulting in a posterior oropharynx detection accuracy of 97.9%, highlighting the effectiveness of the method. Moreover, the model achieved an average precision (mAP50) of 85.7% for uvula detection, indicating good performance across both target regions. These techniques ensure that the model can better generalize the target location information in images, making it more adaptable to real-world data inputs. Additionally, our approach demonstrates the potential to achieve high performance with a smaller training dataset, which is a key advantage when dealing with the time-consuming and costly image collection process in clinical environments. Furthermore, the model’s fast inference speed of 15 ms per image makes it suitable for real-time applications. While data augmentation helps mitigate the limitation of a relatively small dataset, future work will involve incorporating larger and more diverse datasets to improve the model’s generalization ability and extend its application in a wider range of clinical settings.

## 4 Conclusion

This study demonstrated a new flexible robotic system designed to perform OP swab sampling tasks. The sampling robot primarily comprises a flexible series-parallel hybrid manipulator and a visual-tactile fusion module. The series-parallel hybrid mechanism consists of two omnidirectional bendable segments, enabling dexterous pose adjustments for sampling manipulation. The visual-tactile fusion module incorporates a 2D camera to capture an image of the oral cavity after the testee opens their mouth, followed by recognition of the posterior oropharynx position in the xoy plane using a deep learning algorithm. The force sensor within the visual-tactile fusion module detects contact force at the distal end of the OP swab. data from the visual-tactile fusion module is used to control the distal pose of the series-parallel hybrid manipulator for autonomous sampling operations. Preliminary experiments demonstrated that the robotic system is effective for OP swab sampling. Future work will involve integrating protective covers and disinfection measures to prevent potential contamination from viruses.

## Data Availability

The original contributions of this study are included in the Supplementary Material, and further inquiries can be directed to the corresponding author.
